# Interindividual Differences in Cognitive Variability Are Ubiquitous and Distinct From Mean Performance in a Battery of Eleven Tasks

**DOI:** 10.5334/joc.371

**Published:** 2024-05-21

**Authors:** Nicholas Judd, Michael Aristodemou, Torkel Klingberg, Rogier Kievit

**Affiliations:** 1 Cognitive Neuroscience Department, Donders Institute for Brain, Cognition, and Behavior, Radboud University Medical Center, Nijmegen, The Netherlands; 2 Department of Neuroscience, Karolinska Institute, Stockholm, Sweden

**Keywords:** Variability, Cognition, development, intelligence, inter-individual differences, cognitive fluctuations

## Abstract

Our performance on cognitive tasks fluctuates: the same individual completing the same task will differ in their response’s moment-to-moment. For decades cognitive fluctuations have been implicitly ignored – treated as measurement error – with a focus instead on aggregates such as mean performance. Leveraging dense trial-by-trial data and novel time-series methods we explored variability as an intrinsically important phenotype. Across eleven cognitive tasks with over 7 million trials, we found highly reliable interindividual differences in cognitive variability in every task we examined. These differences are both qualitatively and quantitatively distinct from mean performance. Moreover, we found that a single dimension for variability across tasks was inadequate, demonstrating that previously posited global mechanisms for cognitive variability are at least partially incomplete. Our findings indicate that variability is a fundamental part of cognition – with the potential to offer novel insights into developmental processes.

## Introduction

Cognitive performance fluctuates: The same individual performing the same task will demonstrate differences in their performance across trials. Traditional studies of individual differences have implicitly treated this variability as noise or measurement error, focusing instead on averages or sum scores that largely ignore such fluctuations in favor of summary measures such as the mean or total score across trials (Deary et al., 2021; Fiske & Rice, 1955).

Focusing on individual differences in *mean* cognitive performance has borne many fruits, predicting outcomes such as educational attainment, income, health, and longevity (Calvin et al., 2017; Deary et al., 2021). Ignoring cognitive fluctuations is a sensible approach if the information gained is meaningless (i.e., noise) or adds no unique information beyond mean performance (Jensen, 1982). Yet, early historical accounts (Fiske & Rice, 1955; Horn, 1966; Hull, 1943), as well as more recent proposals (Fischer & Bidell, 1998; Thelen & Smith, 1994; Van Dijk & Van Geert, 2014; van Geert, 1991) suggest that cognitive variability is a fundamental behavioral phenotype – playing a functional role in learning (Nesselroade, 1991; Siegler, 2007a), aging (MacDonald et al., 2009) and neurodevelopmental disorders such as attention deficit hyperactivity disorder (ADHD) and autism (Aristodemou et al., 2023; Karalunas et al., 2014; Kofler et al., 2013).

However, in comparison to traditional performance measures (mean or sum scores) empirical work on variability is much less common, and conceptual gaps remain. This scarcity has many causes, but two stand out; *1)* a lack of suitable quantitative methodology and *2)* the necessity of population-scale trial-by-trial data, which until recently remained out of reach. However, several methodological breakthroughs (Asparouhov et al., 2018; Hamaker et al., 2018) and the rapid increase in dense cognitive data (Bignardi et al., 2021; Trull & Ebner-Priemer, 2014) have opened up new opportunities to understand cognitive variability.

The empirical work on variability that *does* exist suggests that cognitive variability provides insights into the dynamics of key developmental periods such as early childhood and old age. In children, differences in cognitive variability predict academic and cognitive performance (Galeano Weber et al., 2018; Judd et al., 2021; Kautto et al., 2023), while in the elderly greater variability is associated with subsequent cognitive decline (Lövdén et al., 2007; MacDonald et al., 2009; Rabbitt et al., 2001). In a clinical context, cognitive variability is a relatively sensitive marker of neurodevelopmental disorders such as autism spectrum disorder (ASD) and ADHD (Aristodemou et al., 2023; Karalunas et al., 2014; Kofler et al., 2013). Although these preliminary studies strongly suggest that cognitive variability offers rich insights into understanding cognitive performance and development, they have almost exclusively focused on a single task or a narrow cognitive domain.

This limited focus has left vital gaps in our understanding of cognitive variability as a novel construct of interest. A fundamental pair of unanswered questions are *1)* whether meaningful individual differences in intraindividual variability are present in all cognitive tasks, and if so, *2)* how these differences in variability relate across tasks in the same individual. In other words, is variability in one task correlated to variability in other cognitive tasks, possibly as a reflection of a single underlying tendency shared across tasks? Or, is cognitive variability a more task specific phenotype?

A better understanding of this structure of individual differences in cognitive variability will give us insights into the underlying mechanisms and likely consequences of this largely neglected phenotype. For instance, the observation of the *‘positive manifold’* in intelligence research (that individual differences in mean performance across tasks is highly positively correlated) and the *‘Big Five’* in personality research (that individual differences in personality can be summarized as varying along five dimensions) has inspired a considerable body of research. Studying the structure of individual differences has shed light on underlying mechanisms, developmental processes, and etiology, as well as their ability to predict various key outcomes (Deary et al., 2021; Keele et al., 1985; Soto, 2019). Variability could manifest as a broader ‘trait’ across a range of tasks, or be more closely associated with features of a specific task. To gain insights into cognitive variability’s ubiquity and structure we must examine it across a range of cognitive tasks.

Plausible mechanisms that would induce domain- or task *specific* variability include differences in expertise – repeated engagement in the same task leads to increased mean performance, decreased variability, and automatization (Li et al., 2004; Lövdén et al., 2020). Some individuals may have more expertise or experience in one task, other individuals in another, leading to modest correlations across tasks. A related but distinct explanation is differences in task strategy. For many cognitive tasks, there are different strategies to achieve better or worse performance, and individuals differ in the extent to which they explore different strategies, versus exploit a given approach (Frank et al., 2009; Siegler, 2007b; Van den Driessche et al., 2019). For instance, individuals use different strategies in how they perform Raven’s matrices depending on the nature of the task (Jastrzębski et al., 2018; Laurence & Macedo, 2023), and even simple numerosity tasks like ‘number line’ show different strategies, with some individuals preferring ‘counting’ and others preferring ‘mental anchoring’, leading to differences in performance properties (White & Szűcs, 2012). Such person-specific strategy use would manifest as weakly correlated individual differences across tasks, whereby an individual may try out different strategies within one task, yet not another, while a different individual could show the opposite pattern.

In contrast, a distinct class of mechanistic explanations are ‘trait-like’ in nature – in other words, they are most simply understood as global mechanisms that would likely affect variability similarly across a wide range of tasks or domains. For instance, neural variability – a broad class of neural measures with differing biological and temporal scales (e.g., neuronal spiking patterns, oscillatory power of local field potentials, time-series of *f*MRI) – has been proposed as one such mechanism fundamental for behavior (Faisal et al., 2008; Garrett et al., 2011; Waschke et al., 2021). Moreover, neural variability is particularly relevant for brain networks engaging in cognitive processing (Mueller et al., 2013). Interindividual differences in neural variability would lead to a pattern whereby people who are more variable on one task are also more variable on (all) other tasks. Other causes of variability likely to affect most or even all tasks similarly include fatigue (Könen et al., 2015; Ratcliff & Van Dongen, 2011), affect (Neubauer et al., 2019), (in)attention (Cohen & Maunsell, 2010; Fukuda & Vogel, 2009), motor variability (Dhawale et al., 2017; Wu et al., 2014), differences in dopamine levels (Cools & D’Esposito, 2011), white matter integrity (Britton et al., 1991; McCormick et al., 2023; Wiker et al., 2023), and locus-coeruleus norepinephrine activity (Aston-Jones & Cohen, 2005; Hauser et al., 2016).

Whether cognitive variability is a more global or task specific phenomenon has contrasting implications on its meaning as a construct, and, in turn how it impacts translational applications such as educational assessment, high-stakes testing, and pharmacological or other interventions for neurodevelopmental disorders. Despite the importance of these questions, to date, no empirical work has thoroughly examined these contrasting intuitions about individual differences in variability.

In this study, we set out to explore the nature of individual differences in intraindividual variability by addressing three urgent questions: *1)* Ubiquity: are interindividual differences in intraindividual variability a consistent property across a battery of 11 cognitive tasks, *2)* Structure: how are individual differences in variability across tasks related, and, *3)* Discrimination: is it distinct from mean performance? To test these we leveraged rich timeseries cognitive data (7,204,127 trials) across a large number (2,608) of children engaging in 11 different tasks on an online mathematical training platform (Judd & Klingberg, 2021). To quantify the dimensionality of variability we use a combination of Dynamic SEM (Hamaker et al., 2018; McNeish & Hamaker, 2020) (to quantify variability within each task) and factor modeling (to examine the quantitative structure of variability across tasks), which together allow us to use model comparison techniques to compare and contrast competing explanations. Doing so allows us to investigate, for the first time, the structure of this often-neglected property of cognitive performance.

## Results

**Figure 1:**
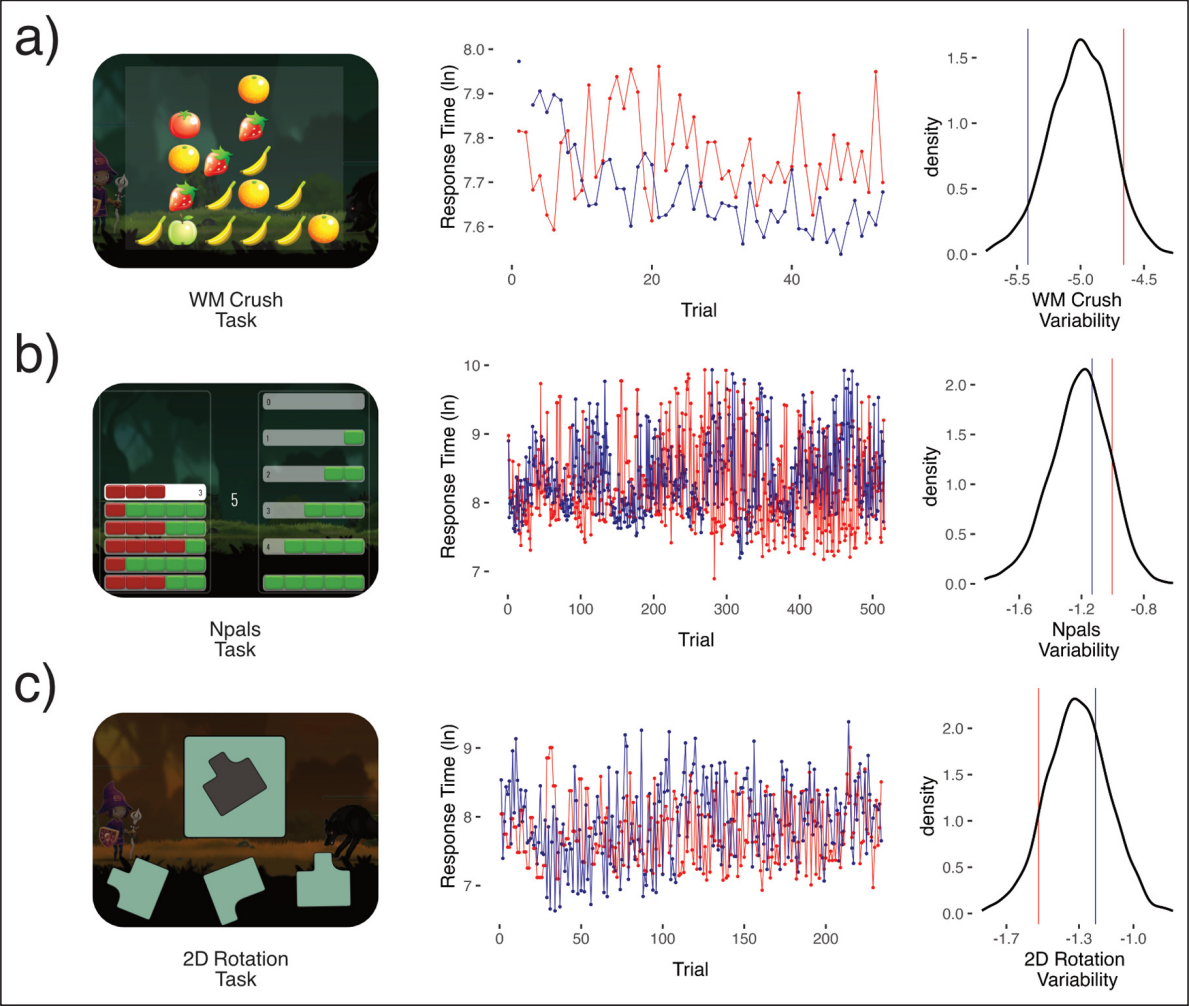
Shows three of the eleven cognitive tasks used **a)** visuospatial working memory crush, **b)** Number pals, and **c)** a 2D Mental Rotation task. For illustration purposes we randomly sampled one subject from the bottom 10% of the WM Crush variability distribution (in blue), and one subject from the top 10% (in red). The same subjects were then plotted for b) number pals and c) 2D Mental Rotation. We then plotted their logged response times by trial number, limiting the x-axis to the sample mean trial number of that task. Next, density plots show the distribution of our measures of cognitive variability factor scores extracted from dynamic SEM and the relative position of the two subjects (blue & red). Cognitive variability is modeled using a log-linear model, to preclude negative values. Note, however, that the logarithm of the variance can be negative as seen in the x-axis of the density plot.

We included data from 2,608 children between the ages of six and eight who completed cognitive tasks for up to eight weeks on a mathematical training app (Judd & Klingberg, 2021; Nemmi et al., 2016). This resulted in slightly over 7 million correct response times across eleven distinct cognitive tasks (Figure 1 shows examples from 3 tasks). Each trial reflected the amount of time in milliseconds that a child took to correctly respond to a stimulus in one of the 11 tasks. From these trials we derived a model-based estimate of cognitive variability per task using a Dynamic SEM (see Methods for more details). This modeling technique allowed us to quantify individual differences in residual variability after adjusting for other sources of variance in the timeseries of trials. To complement our investigation of cognitive variability, we also examined the mean correct difficulty level (called here “mean performance”) for each of the tasks in the same sample as a reference. Our key question of interest was the relationship of variability estimates between tasks, and how they inform our understanding of the underlying causes and possible consequences of variability.

A crucial step in Dynamic SEM (DSEM) is to establish the presence, or absence, of random effects – in other words, do people *differ* meaningfully on parameters in the model, including our key parameter of interest, variability. This step is crucial, because if variability does not differ systematically between people, it may be more tenable to ignore it as intrinsic, system-level noise irrelevant to human cognitive performance in a broader sense. To test this key question, we used model selection (difference in Deviance Information Criterion, or DIC values, a model information criterion that penalizes complexity) by comparing a model with a random effect for the residual variability parameter, to a model without. Doing so we observed that a model with random effects for residual variability was overwhelmingly favored for each task (SI Table 1). This demonstrates that individual differences in intraindividual variability are ubiquitous across the 11 tasks studied here. Intriguingly, these response time variability estimates only correlated modestly with mean response time estimates for each task (SI Fig 3). This illustrates the ability of DSEM to successfully separate these distinct concepts along with offering evidence that variability is a distinct facet of the cognitive phenotype. Moreover, the large sample size and trial number meant that DSEM variability estimates were also observed to be highly reliable within tasks (SI Table 2), allowing us to use these estimates in further analyses to examine how they were associated across tasks.

**Figure 2:**
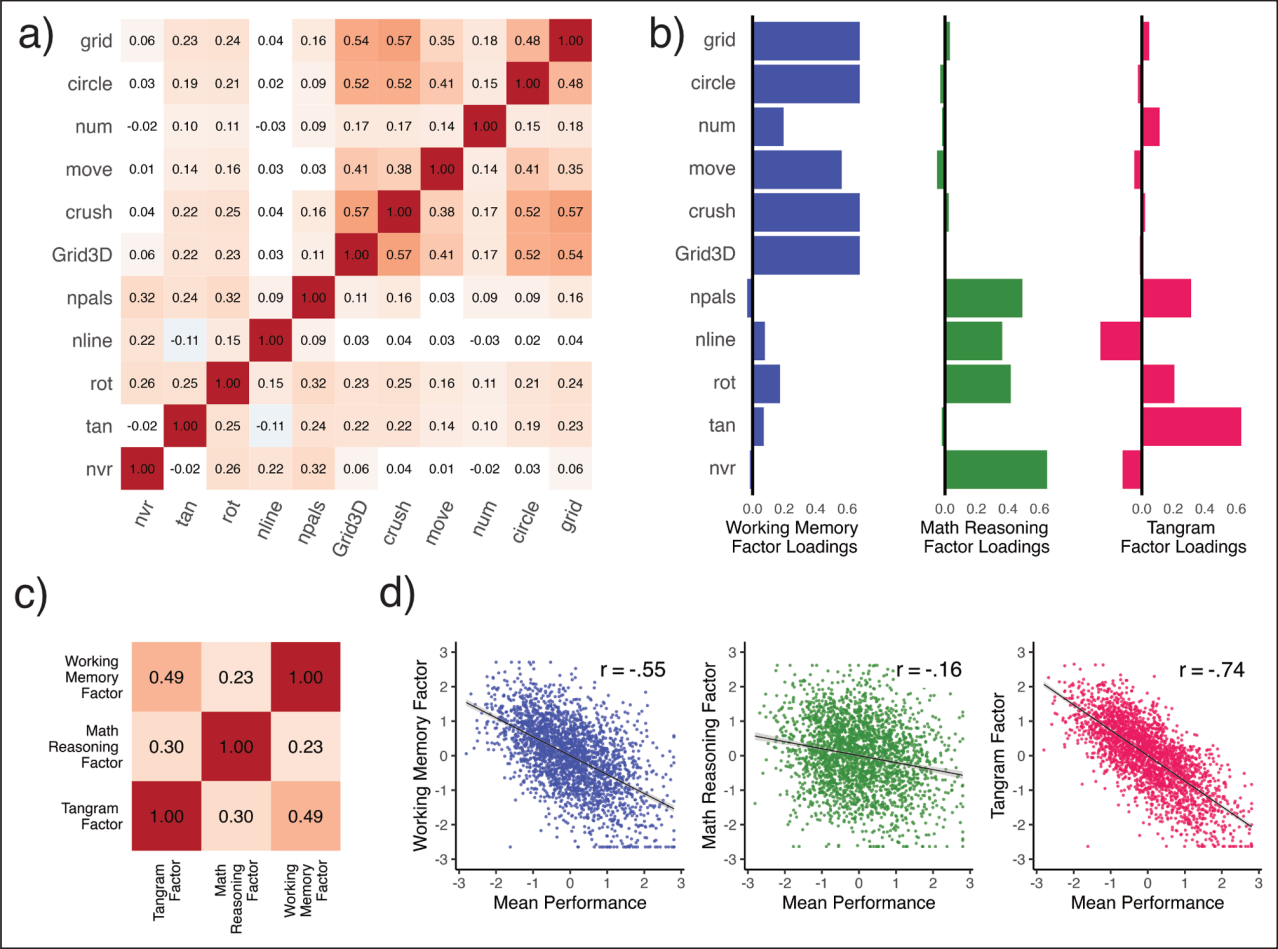
Illustrates interindividual variability measures (n = 2,608) in response time from Dynamic SEM across 11 cognitive tasks. Panel **a)** shows a Pearson r correlation plot with only significant (Holm’s multiple comparison correction) associations colored, while panel **b)** shows the loadings (SI Table 4) for the first three factors in an exploratory factor analysis. The first factor (‘Working Memory’) captured 22% of the variance, the second (‘Mathematical Reasoning’) accounted for 9%, while the last (‘Tangram’) accounted for 6% of the variance. Panel **c)** shows the correlations between these three factors while **d)** illustrates the relationship of each variability factor with a single factor of mean level (i.e., *Mean Performance*).

After demonstrating reliable and substantial individual differences in intraindividual variability in all 11 cognitive tasks, next we examined how these differences in variability relate across tasks. To accomplish this, we extracted the DSEM derived variability factor scores – reflecting interindividual differences in variability – for each of our 11 tasks. Doing so we found response time variability between tasks to be mostly positively correlated (Figure 2a). However, the average magnitude of these correlations was modest (Mean Pearson’s r = .19), suggesting that on average those who were more variable on one task, were also more variable on others – but only weakly so. In fact, twelve pairwise correlations were not significantly different from zero, despite the considerable sample size and high reliability of the parameter estimates. To ensure this relatively weak pattern of correlation was not a feature of either the population or task battery, we also examined the correlation between mean performance across all tasks. Here, in contrast, we observe the omnipresent ‘positive manifold’ (McGrew, 2009) – mean performance on each of the 11 tasks was correlated strongly (Mean Pearson’s r = .57) and positively across the board (SI Fig 4b).

Additionally, to make sure that our weak between-task variability correlations are not an artifact of differing temporal sampling rates (Table 1), we correlated absolute dissimilarity in median RT between tasks with our between-task variability correlations. This showed dissimilarity in median RT task length to be unrelated (Pearson’s r = –.24, p > .05) to the strength of between task variability correlations, suggesting that at this timescale at least, differences in response time did not induce artificial large differences in variability. This pattern of weaker correlations for variability relative to mean performance was also observed through a dimension reduction approach: extracting a first factor across all tasks explained only 25% of the variance in cognitive variability measures, but 58% of the variance of the mean performance measures. Next, we examined the underlying structure of cognitive variability in more detail.

**Table 1:** Task Overview – number of trials and response time (RT) descriptives.


TASK	TOTAL TRIALS	MEDIAN TRIALS PER CHILD	RT MEDIAN	RT IQR	RT MIN	RT MAX

WM_3dgrid (3DGrid)	203738	78.12	7502.75	1502.50	4088.0	12344.00

WM_circle (circle)	155878	59.77	7604.50	1406.62	4125.0	13004.00

WM_crush (crush)	243511	93.37	7356.75	1547.50	4100.0	10830.00

WM_grid (grid)	430708	165.15	7868.00	1319.00	3798.0	15205.00

WM_moving (move)	85640	33.34	7508.00	1471.50	4149.0	12348.50

WM_numbers (num)	98300	37.94	8017.00	1405.00	5754.0	23362.50

Number pals (npals)	2398463	919.66	3659.50	640.25	1846.0	8139.00

Numberline (nline)	1833909	703.19	6102.50	2201.38	2617.0	15905.00

Non-verbal reasoning (nvr)	548101	210.16	6780.75	1655.25	4088.0	12344.00

Rotation (rot)	1032751	395.99	3420.75	1118.38	4125.0	13004.00

Tangram (tan)	173128	66.38	7502.75	1502.50	4100.0	10830.00


### Testing the dimensionality of cognitive variability

Above, we found that despite a highly powered sample with reliable measures of variability, we observed relatively weak, albeit mostly positive correlations between tasks. To better understand the nature of this correlation pattern, and thus cognitive variability itself, we explored the structure of correlations among the tasks. A relatively simple model to test is a unidimensional factor model, which captures the hypothesis that individual differences in variability across tasks are plausibly explained by a single underlying factor and that any residual variance is task specific. To formally test this hypothesis, we fit a confirmatory factor model with a single underlying factor for *cognitive variability* (SI Fig 2a). This model fit poorly (χ^2^ = 1058.4, df = 44, p < 0.0001, RMSEA = 0.094 [0.089, 0.099], CFI = 0.83) demonstrating that a single underlying mechanism driving individual differences in intraindividual variability across a range of tasks is inadequate for the structure we observe.

To further investigate individual differences in variability we considered two more complex confirmatory models. Although there is no substantive research examining the structure of cognitive variability across a wide range of tasks, some authors have suggested that cognitive variability and mean performance may be two sides of the same coin (Galeano Weber et al., 2018), suggesting the same measurement model should fit both domains. To test this hypothesis, we constructed an *a priori* hierarchical model with three subdomains (Math, WM, and Spatial, shown in SI Figure 2b) which did not converge for cognitive variability due to negative variance for the math subdomain (*i.e.*, a Heywood case). Notably, this model fit the *mean performance* measures well (SI Fig 5b; SI Table 3), suggesting both the domain-based grouping and tasks are defensible – our tasks can replicate the mean performance literature (McGrew 2009). Finally, we then fit a hierarchical model with a single working memory subdomain. This model fit moderately well – an improvement over both the unidimensional model and the *a priori* mean performance-inspired three-factor model, but still not sufficiently well (SI Fig. 5a; χ^2^ = 517, df = 43, p < 0.0001, RMSEA = 0.065 [0.060, 0.070], CFI = 0.92). Due to the lack of adequate fit for cognitive variability, we did not attempt to test a latent correlation between variability and mean performance, shifting to an exploratory approach instead.

### Exploring the structure of cognitive variability

Above we found robust evidence that *cognitive variability* is both qualitatively and quantitatively distinct from mean performance, and showed that multiple plausible models did not fully capture the dimensionality of variability. To further explore the underlying structure, we switched to an exploratory factor analysis framework. Doing so, we observed that an exploratory three-factor solution fit cognitive variability well (see Methods). The overall correlation structure and pattern of loadings is shown in Figure 2.

The first factor (which we call the ‘*WM-factor’* as it was dominated by high loadings on WM tasks), explained 23% of the variance and had close to or near zero loadings with the rest of the tasks (Figure 2b; SI Table 4). The second factor (*mathematical reasoning*) explained 9% of the variance, with substantial positive loadings with number pals, number line, rotation, and non-verbal reasoning, yet near-zero loadings across all WM tasks and tangram. Lastly, the third factor (*Tangram*) explained only 6% of the variance yet it was also the only factor that the tangram task loaded on (.63). Intriguingly, the Tangram factor was also the only factor that showed non-trivial *negative* factor loadings. Mental rotation showed an intriguing pattern, as it was the only task to show reasonable positive loadings across all three factors.

We found these three variability factors to correlate modestly (Figure 2c) with each other. Intriguingly they showed distinct relationships with *mean performance* (Figure 2d). To examine this question, we extracted factor score estimates from the first exploratory ‘mean performance’ factor, and related it to each variability factor in turn (Figure 2d). The first factor, *working memory*, was substantially correlated with mean performance (Pearson’s r = –.55, p_HOLM_ < .001), while the mathematical reasoning factor was only weakly correlated with mean performance (Pearson’s r = –.16, p_HOLM_ < .001). Contrary to our expectations, we found that the third, ‘Tangram’ variability factor showed the strongest association with mean performance (Pearson’s r = –.74, p_HOLM_ < .001). Williams’s tests determined all of these correlations to be significantly different from each other (*p’s*_HOLM_ < .001), further supporting both the distinction between variability and mean performance, as well as the specificity of distinct variability factors in our sample.

## Discussion

Across trial-by-trial data from 2,608 children, we find *cognitive variability* to be a distinct and ubiquitous behavioral phenotype. Individual differences in intra-individual variability were present across all 11 different cognitive tasks. Crucially, variability is distinct both qualitatively and quantitatively from *mean performance*, demonstrating some domain general properties, but showing substantially more task heterogeneity than mean performance. Despite the high within-task reliability, homogeneous task implementation, and large sample size, a substantial portion of tasks showed small or absent correlations in variability, suggesting unique, task specific properties contributing to the overall structure. These findings demonstrate that a variability perspective can offer a rich, novel cognitive phenotype derived from existing data. It also provides evidence that single cause explanations – such as attention, fatigue, dopamine – must be at least partially incomplete.

The absence of strong positive correlations across all tasks for cognitive variability is in stark contrast with mean performance measures (Deary et al., 2021; McGrew, 2009). Crucially, it makes global mechanistic accounts, which would suggest that greater variability on one task would be associated with more variability on all other tasks – less plausible or at least incomplete. Although we did observe some shared variance in cognitive variability through dimension reduction techniques, the pattern of associations observed suggests it is unlikely that any single individual mechanism, be it fatigue, affect, (in)attention or neural differences (e.g., dopamine & white matter integrity) could fully explain all interindividual differences in variability. In other words, although our findings do not allow for inferences about the role of any individual mechanism, it does provide indirect evidence suggesting multiple, partially distinct underlying causes are likely at play. This was further confirmed through a lack of subdomain structure in mathematics or spatial abilities, in contrast with *mean performance* measures (McGrew, 2009). However, we did observe that variability estimates from different working memory tasks show some substantial degree of covariation (~.5 correlations between tasks). Moreover, there was a limited subdomain structure found in both our confirmatory and exploratory analyses accounting for 23% of the variance. This suggests that although some drivers of variability may play a role in multiple tasks, it is highly likely multiple mechanisms are at play. Our findings suggest the need for a hybrid explanatory framework allowing for both task-specific and more general mechanisms driving variability.

While working memory showed strong within-domain variability correlations, these tasks had very weak and non-significant correlations with both number line and non-verbal reasoning – again in stark contrast with our findings here for mean performance, as well as the mean performance literature more generally (Ackerman et al., 2005; Gathercole et al., 2004). Working memory is so central to *mean performance* it has been theorized to be “*the mechanism”* causing interindividual differences in cognition (Kovacs & Conway, 2016). One potential reason for this diverging pattern between variability and mean performance is that both tasks (Numberline and NVR) have high complexity, allowing more strategy shifting as children progress through different levels. For example, number line progresses across different types of mathematics (e.g., addition, division, fractions, etc.) – each requiring different strategies, and, in turn, increasing task-specific variance. Cognitive variability’s low correlation between tasks – despite high within-task reliability, homogeneous task implementation and a large sample size – leads us to conclude the absence of certain task relationships and subdomains is most likely due to them being qualitatively different from each other.

Across the board, we observe robust negative correlations between our cognitive variability factors and mean performance, in line with previous single task findings for WM (Galeano Weber et al., 2018), simple reaction time tasks (Kofler et al., 2013), language (Kautto et al., 2023) and matrix reasoning (Rabbitt et al., 2001). This indicates that children with worse *mean performance* show much more *cognitive variability*, while higher performing children are more consistent across trials. In practice, this means that one-off testing (e.g., standardized testing) is more likely to misestimate mean performance in lower ability children, potentially leading to longer-term consequences when used for educational stratification or diagnostics. Notably, even if a child with high variability were to score relatively highly in a high-stakes setting this is unlikely to have commensurate *benefits* – being tracked into a higher level than one’s ability – can lead to disillusion, dropout, and worse long-term academic outcomes (Abdulkadiroğlu et al., 2014; Duflo et al., 2011; Valentine et al., 2004).

This study is the first to examine the underlying structure of variability in a large sample with diverse cognitive tasks. However, there are of course limitations and outstanding questions left unanswered. We focus on a particular age range (6–8-year-olds), putting some a priori limitations on generalization to other developmental stages and samples. Moreover, the participants predominately come from Sweden and do not reflect a comprehensive sampling of the global population. While the application was tailored for teachers and a classroom setting, we did not exclude based on this criterion, this means a small subset of children in our sample completed the task at home. Finally, our analytic approach using DSEM is powerful and flexible, but other quantitative approaches (e.g., drift-diffusion model or exgaussian models, e.g. (Epstein et al., 2023; Schmiedek et al., 2007)), will have a complementary set of strengths and weaknesses in uncovering key parameters. For instance, a drift-diffusion modeling strategy would allow the separation of motor preparation and execution (i.e., non-decision time) component from a cognitive one (Ratcliff, 2006), at the expense of time-series modeling.

Exactly how motor variability might interplay with cognitive variability remains an outstanding question. There is strong experimental evidence that motor variability is involved in learning, an inherently cognitive process (Olveczky et al., 2005; Wu et al., 2014). Motor variability – thought to be an unwanted consequence of a noisy nervous system (Dhawale et al., 2017) – would increase the similarity of interindividual differences across tasks leading to larger correlations between tasks. Previous findings have found variability in simple response time tasks to be predominantly cognitive (when controlling for motor processes) (Epstein et al., 2023; Karalunas et al., 2014; Schmiedek et al., 2007), and crucially linked cognitive variability in more complex cognitive tasks to disparate outcomes such as cognitive decline (Lövdén et al., 2007; MacDonald et al., 2009), fluid intelligence (Galeano Weber et al., 2018; Rabbitt et al., 2001), inattention/ADHD (Aristodemou et al., 2023; Kofler et al., 2013), and academic achievement (Judd et al., 2021). Nevertheless, our estimates most likely have a limited motor component, yet the lack of a large common factor across tasks suggests it cannot fully explain the patterns we observe. Moreover, each cognitive variability factor was related to mean performance, and two variability factors were more strongly correlated with mean performance than with each other, suggesting our interpretation of variability as a *cognitive* phenotype is warranted. Lastly, all of our tasks were designed to tap into high-level cognitive abilities and were administered within the same setting, response profile, and reward structure, leaving open (even) more high-dimensional patterns.

Another intriguing avenue is in exploring the pattern of interindividual differences we found between cognitive tasks. Task heterogeneity hints towards the possibility of cognitive variability being a phenotypically differentiating tool, that is, variability in one task (e.g., math) might offer insights entirely separate from variability in another task (e.g., WM). This could be explored one of two ways, first *1)* by identifying influences of global and task specific causes, and secondly *2)* determining the relationship between mean performance and variability over time. To be able to tease apart these relationships more dense longitudinal designs with detailed analysis focused on the processes that give rise to variability are needed (Siegler, 2007b). For instance, prior longitudinal work has explored how variability is related to cognitive aging, emotional dynamics, and motor performance (Janssen et al., 2016; MacDonald et al., 2003; Ram et al., 2014; Reitsema et al., 2023). Crucially, trial-level data is necessary to identify temporally specific causes – evidence suggests distinct types of variability operate between short (moment-to-moment) and long timescales (e.g., session-to-session & day-to-day) (Galeano-Keiner et al., 2022). For instance, moment-to-moment variability could identify a period of intense focus or flow, while day-to-day variability could highlight longer-term fluctuations in sleep or affect (Neubauer et al., 2019; Siegler, 2007a).

Our results suggest that a few different cognitive tasks should be used when studying variability since findings in one task will not necessarily be informative of variability in another task. For example, future designs could modify dopamine levels experimentally with methylphenidate (Pertermann et al., 2019) while tracking task specific strategies on a trial-by-trial basis across a few disparate cognitive tasks. This design would allow the isolation of dopamine’s influences on cognitive variability, while its time-series nature would also allow insight (Williams et al., 2021) into learning processes such as how early variability could cause differences in mean improvement (e.g., exploitation vs. exploration) (Frank et al., 2009; Gopnik et al., 2017; Van den Driessche et al., 2019). These are just a few examples of how a variability approach can offer novel questions and potentially unique insights into cognitive performance.

Here we report *cognitive variability* to be qualitatively and quantitatively distinct from *mean performance* across eleven tasks with over seven million trials. Variability was both substantially heterogeneous across tasks and highly reliable within tasks. Some relationships we expected a priori, such as that between WM and reasoning, were intriguingly absent. Although we did find some common variance across tasks it was not enough to support a singular common cause explanation. Future work should attempt to disentangle these different causes of variability across tasks, as it is likely to offer novel insights into developmental processes underlying human cognitive performance.

## Methods

### Study description

We included children between the ages of six to eight who had completed a mathematical training app (Vektor) with number line based exercises that have been shown to improve math in 6-8-year-old children (Judd & Klingberg, 2021; Nemmi et al., 2016). We focused only on children with 25 sessions enrolled in the mixed cognitive training plan since it had the widest task diversity. This resulted in 2,608 children with a total of 7,204,127 trials across eleven cognitive tasks over eight weeks.

### Cognitive Tasks

The battery in Vektor consists of 11 distinct tasks which, from a traditional cognitive psychological perspective, can be grouped into four classical domains; mathematics, working memory (WM), spatial abilities, and nonverbal reasoning (SI Fig. 1; Video: https://osf.io/vhcd5). All tasks had a set of guided trials at the start to help children figure out the task, these were all removed for our analysis.

Out of eleven tasks six were WM tasks; five of which were visuospatial-WM. These five all followed a very similar task design where dots were presented in different locations on a stimuli grid and needed to be repeated back in the correct order on a touch screen (SI Fig. 7). The tasks varied in their spatial layout to make them appealing to children. Difficulty was automatically adjusted by increasing the span of items. For example, the task ‘grid’ displays a 4-by-4 grid on which stimuli become highlighted while WM circle has 10 stimuli arranged in a circular layout. One WM task was conceptually different (WM numbers), this was a digit span backwards task in which Arabic numerals were presented and then needed to be repeated back in reverse order. Due to the age range studied, we considered it to also be part of the mathematics domain allowing it to cross-load on the mathematics subdomain (SI Fig. 2b).

Since the purpose of Vektor was to improve children’s mathematics, roughly half the time was spent on two mathematical tasks. Both tasks (i.e., Number line & Number pals) were based on a visual representation of a mental number line. The first task (numberline), had a visual number line where the child had to use their index finger to drag (left to right) it to the correct position corresponding to an Arabic numeral. The difficulty was first increased by removing contextual cues (e.g., ticks on the numberline) and later by introducing new mathematical concepts (e.g., addition, division, fractions, etc.). The second task (Npals), consisted of bars on the right side of the screen that were partially filled number lines. The participant was then instructed to pull the correct bar (that is, the one with the right number of units to fit) from multiple options on the right-hand side. The difficulty was moderated by bar length, the replacement of bars with Arabic numerals, and increasing the sum from 5 to 10 and then 15.

The non-verbal reasoning task consisted of sequential ordering tasks in which participants viewed a sequence of tiles with spatial patterns and had to choose the correct image to fill the blank in sequence (Bergman Nutley et al., 2011). The difficulty was increased by introducing additional stimulus dimensions (colors, shapes, and numbers of dots) on which the stimuli should be compared. Lastly, the spatial domain consisted of two tasks: 2D mental rotation & tangram. For 2D mental rotation, children were presented with a silhouette of a target image. Underneath the silhouette were multiple options that had to be mentally rotated, only the correct target image fit the silhouette. Difficulty was modified by increasing item complexity. The tangram task also had a black silhouette yet the items presented were all different pieces of the silhouette which needed to be manually rotated and put in the correct place. Difficulty was increased with item complexity and additional pieces (i.e., items).

### Mean Performance measures

For each task, we computed the mean correct level across all trials as our *mean performance* measure. For the WM tasks level corresponded to the number of items to be held in memory (i.e., span) for the other tasks level represents a difficult index that increases with task progression. Our mean performance measures showed high correlations across tasks regardless of domain (SI Fig. 4b) – in line with the well replicated finding of a positive manifold across cognitive tasks.

### Variability measures

For each cognitive task, we fit a dynamic structural equation model (DSEM) on response times using MPLUS (version 8) with 20,000 iterations (Asparouhov et al., 2018). Only response times from correct trials were included. Response time was measured in milliseconds as the time between the end of task presentation and the last response. Before each DSEM model fitting procedure, we log transformed response time (to improve distributional characteristics) and recoded outliers as missing (to limit outsized impact). Outliers (~1.5% of total trials) were identified as values more than 1.5 times the interquartile range within each task on the total population from the quartiles of a boxplot (i.e., Tukey method).

Some of the tasks were designed in a way in which difficulty was increased by adding stimuli, notably, this affected all the WM tasks and the tangram task. This task feature – an inherent property of WM tasks—forces a dependency between ability and response time. In WM tasks trials with larger spans take longer to respond to. To correct for this, we divided response time by the number of items presented. This was done before log transformation, outlier treatment, and further modeling.

DSEM has many benefits and is ideal for modeling variability (Hamaker et al., 2018; McNeish & Hamaker, 2020). Most importantly DSEM can account for within-subject trends and autoregression. We modeled four parameters of interest within and between-subjects; *1)* the mean response time, *2)* residual variability of response times, *3)* inertia (trial carryover parameter), and *4)* the trend (i.e., improvement). Supplementary Figure 3 illustrates the correlations of the between subject parameters for each DSEM fit.

All of the cognitive tasks converged and fit well according to DSEM model diagnostic checks (see https://osf.io/z53an/). To see if there is meaningful variability, we fit DSEMs with the interindividual variance of the residual variability parameter constrained to 0 and compared its fit to the full DSEM model using the DIC for each task. We found the fit was better with the addition of a variance parameter for all cognitive tasks (SI Table 1). We then tested three different scale reliabilities (.5, .7, & .9) using the mean trial length as the number of occasions, across the board we found high trial variance reliability even with low (i.e., .5) scale reliability (SI Table 2) (Du & Wang, 2018).

Lastly, we extracted variability factor scores from each DSEM and used them as our measure of *cognitive variability* for each cognitive task. This resulted in one measure per task of inter-individual differences in response time variability. Previous work has suggested that variability at different timescales (e.g. trial to trial versus occasion to occasion, (Galeano-Keiner et al., 2022)) may be only weakly correlated across individuals. As such, we wanted to ensure that differences in median RT’s between tasks (Table 1) were not governing differences in variability. To test this question, we computed pairwise dissimilarities as the absolute Euclidean distance between median RT using the daisy function in the cluster package (Maechler et al., 2023). The lower diagonal of this dissimilarity matrix was then vectorized and correlated with a vectorized variability correlation matrix (Figure 2a). If similarity in temporal resolution between tasks is in fact the source for between task variability correlations this would be reflected by a significant negative correlation. Conversely, if more temporarily dissimilar tasks correlated more with each other, we would expect a positive correlation. Lastly, if differences in median trial response length is unrelated to the strength of variability correlations, we would expect this relationship to be non-significant and around zero.

### Confirmatory factor modeling

To test the factor structure of cognitive variability and mean performance measures we used confirmatory factor analysis (CFA). For each measure, *1)* a single factor was fit, *2)* then an *a priori* hierarchical factor with three sub-domains (WM, Math & Spatial) was used, and, if neither fit *3)* a hierarchical factor with only WM as a subdomain (SI Fig. 2). The second model had ‘num’ a WM digit span task cross loading on both the mathematics and WM subdomains.

Models were fit in R (version 4.2.2) with the lavaan package (version 0.6–15) using robust maximum likelihood (Rosseel, 2012). Missing data (cognitive variability = .2%; mean performance = .2%) was handled using full information maximum likelihood under the assumption of missing at random (Enders & Bandalos, 2001). We assessed model fit using the comparative fit index (fit > 0.95) and the root-mean-square error of approximation (fit < 0.08) (Hu & Bentler, 1999). All results are reported in fully standardized units (mean = 0, SD = 1).

### Exploratory factor modeling

Since our confirmatory approach did not fully capture the structure of variability, we proceeded to use exploratory factor analysis for the cognitive variability measures using the psych package (version 2.3.3) (R Core Team, 2014; Revelle, 2018). To determine the optimal number of factors we used parallel analysis – a method for determining the number of factors by comparing the empirical eigenvalues to that of random data. In our sample parallel analysis was inconsistent between a three and four factor solution, to empirically test this we looped the analysis 100 times with the first 100 seeds. We found parallel to prefer a 4-factor solution 58% of the time and a 3-factor solution 42% of the time. Upon examining the output, we decided to use a 3-factor solution for the following reasons; *1)* the fourth factor was uninterpretable, *2)* very little explained variance (i.e., 3%) in the fourth factor, *3)* the fourth factor was near the cutoff (SI Fig 6) and *4)*, three factors were needed to achieve moderate positive loadings from all tasks. Importantly, the loading pattern was similar for the first three factors regardless of whether a three or four factor solution was used. Therefore, we decided to report and interpret a three-factor solution with the defaults (i.e., oblimin rotation) from the ‘fa’ function (Figure 2b).

## Data Accessibility Statement

Data, code and model fits to replicate the analysis are openly available (https://osf.io/z53an/). Trial-by-trial data to fit DSEM models could not be publicly released for privacy concerns.

## Additional File

The additional file for this article can be found as follows:

10.5334/joc.371.s1 Supplementary Information. Supplementary Figures 1–7 and Supplementary Tables 1–4.
