# Supplementary material for: Interindividual Differences in Cognitive Variability Are Ubiquitous and Distinct From Mean Performance in a Battery of Eleven Tasks
Source: J Cogn. 2024 May 21;7(1):45. doi: 10.5334/joc.371 (PMC11122693; doi:10.5334/joc.371)
Supplement: Supplementary Information. — Supplementary Figures 1–7 and Supplementary Tables 1–4. [file joc-7-1-371-s1.pdf]

Supplementary Information

Cognitive variability is ubiquitous and distinct from mean performance across eleven tasks with over 7 million trials

Lifespan Cognitive Dynamics Lab
https://lifespancognitivedynamics.com

Nicholas Judd
Micheal Aristodemou
Torkel Klingberg
Rogier Kievit

nickkjudd@gmail.com
https://njudd.com

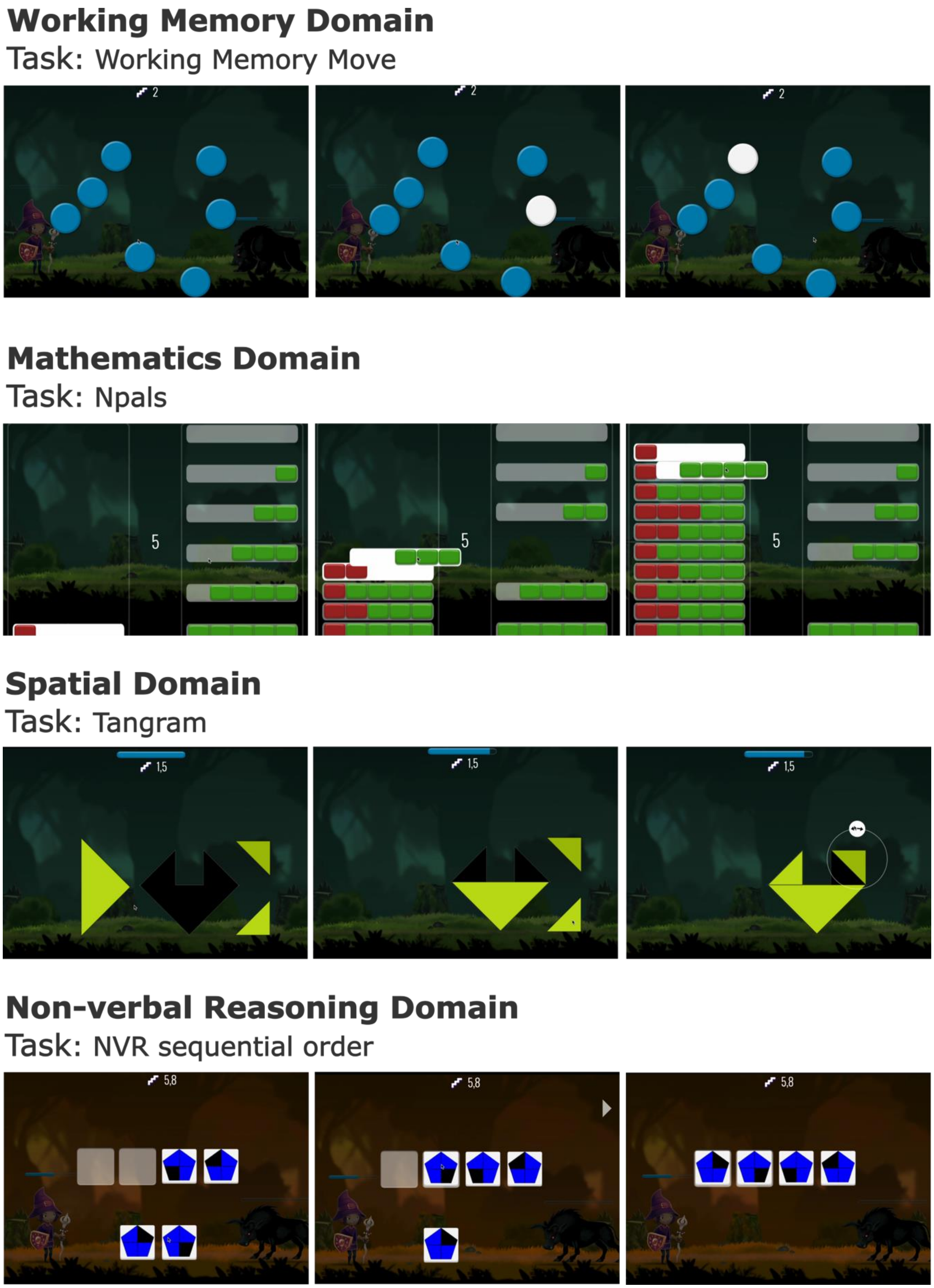

Supplementary Figure 1 An overview of four selected tasks representing each domain; Working memory, Mathematics, Spatial and Nonverbal Reasoning. The images show task progression. For more task images see SI Fig 1 in Judd & Klingberg 2021.

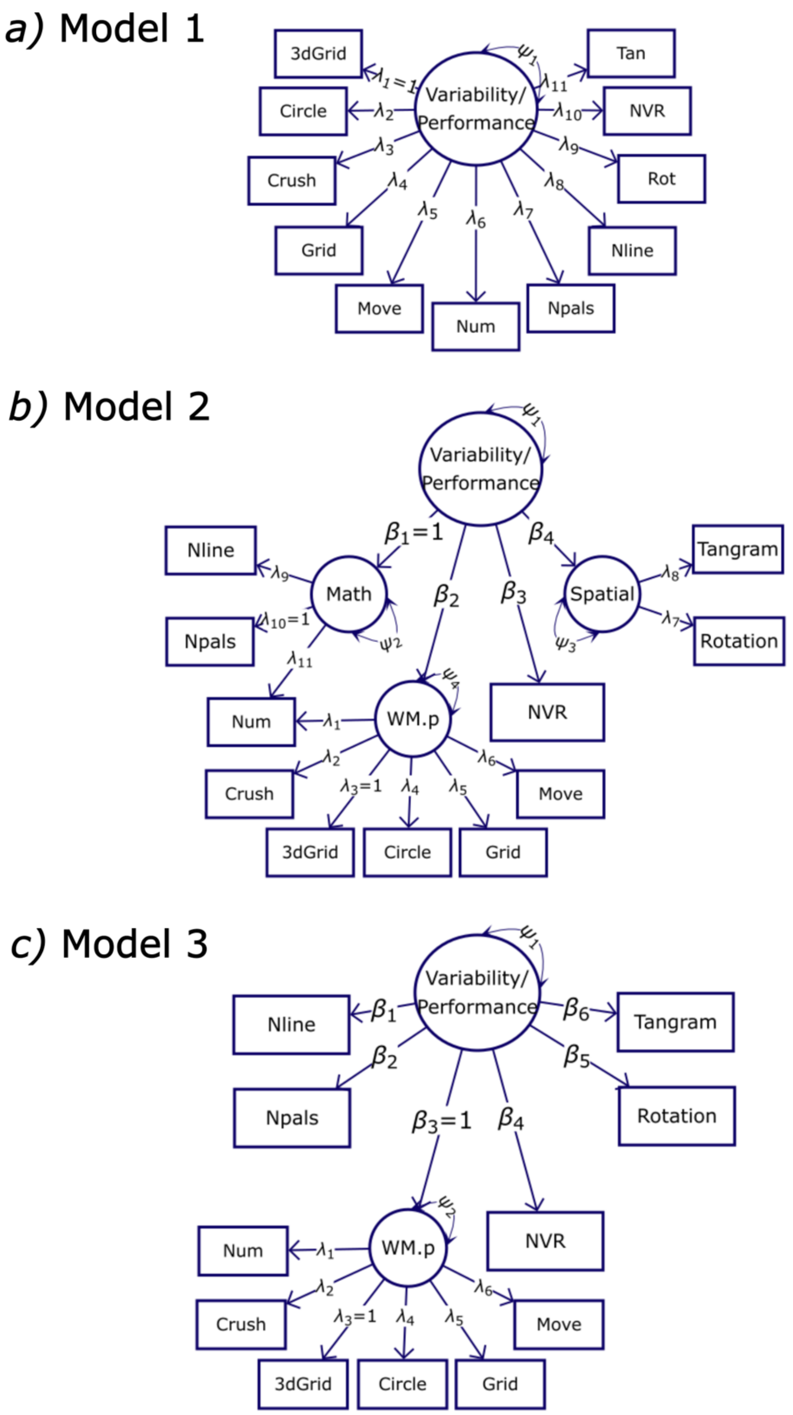

Supplementary Figure 2 An illustration of the three confirmatory factor analysis (CFA) used to test the factor structure of *cognitive variance* and *mean performance*. If an earlier model fit, we did not continue. Model 1 *a)* shows a single factor solution where all the tasks load on one higher order latent cause. Model 2 *b)* shows an *a priori* hierarchical factor structure with subdomains hypothesized from the intelligence literature on *mean performance*. Lastly, Model 3 *c)*, is a working memory only hierarchical factor structure. We found *mean performance* measures to best fit Model 2 while *cognitive variance* adequately fit Model 3 (see SI Table 3).

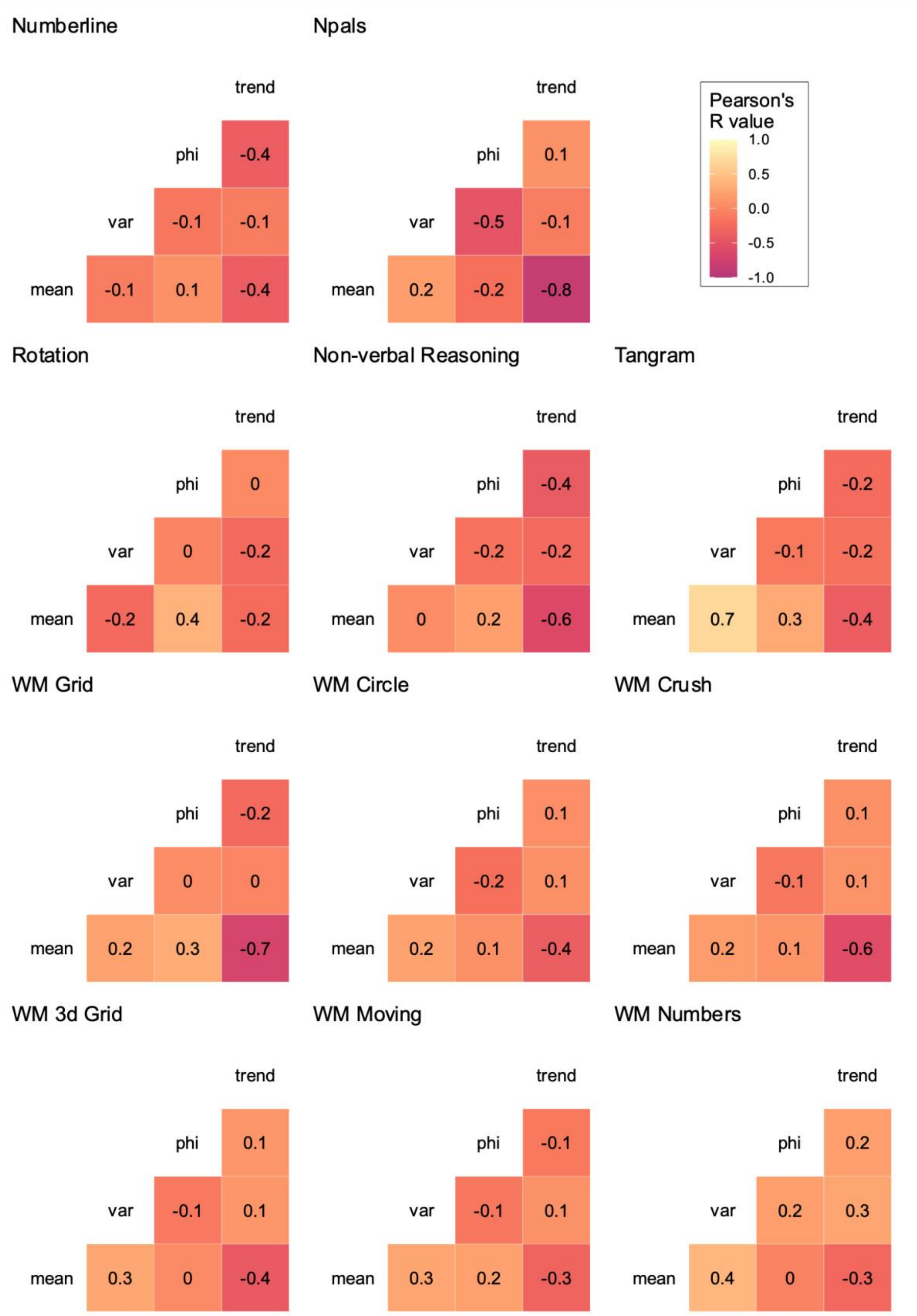

Supplementary Figure 3 Standardized (STDYX) between subject correlation plots for the four parameters fit (mean, variance, phi and trend) in the DSEM per cognitive task. Mean represents the subject's average response time, var indicates the variance around this average. Phi is the inertia parameter which allows trial carry over effects. Trend shows the subjects improvement in the task. For equations and a graphical model representation see (Aristodemou, Rommelse, and Kievit, 2023).

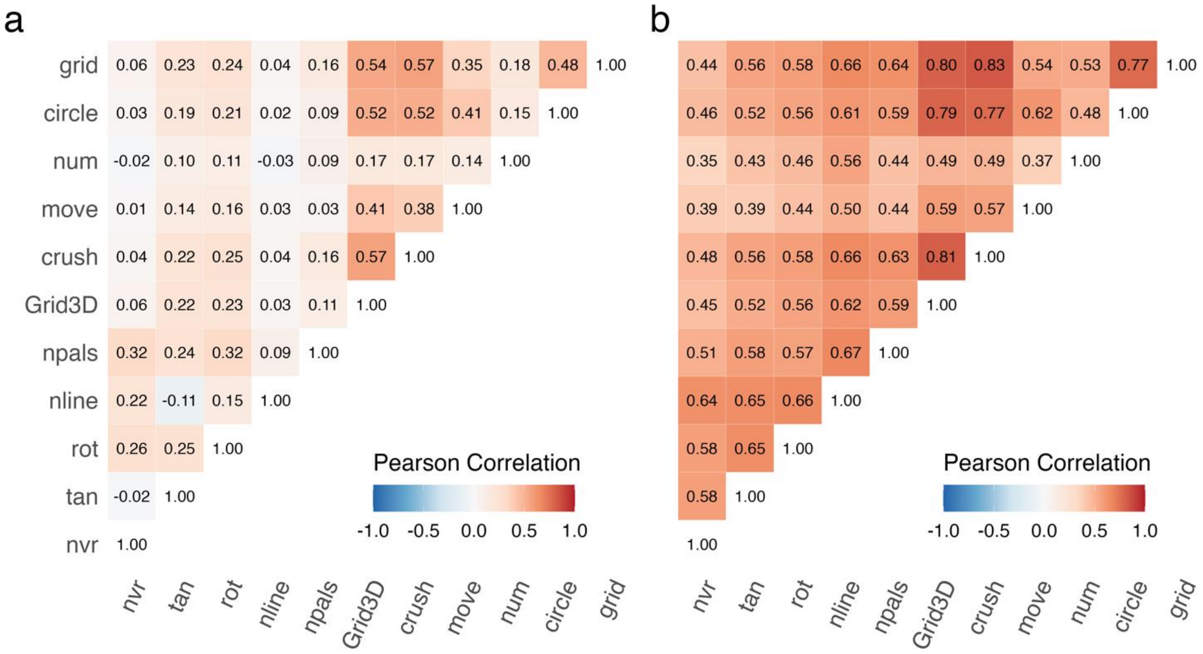

Supplementary Figure 4 Pearson's R correlation plots (n = 2,608) of ***a)*** *variability* measures and ***b)*** *mean performance* measures across 11 cognitive tasks. Interindividual variability measures are extracted by fitting a DSEM per task on all correct response times while *mean performance* measures are the mean correct level over the course of 8 weeks. All associations are colored, regardless of significance (p-values are unadjusted).

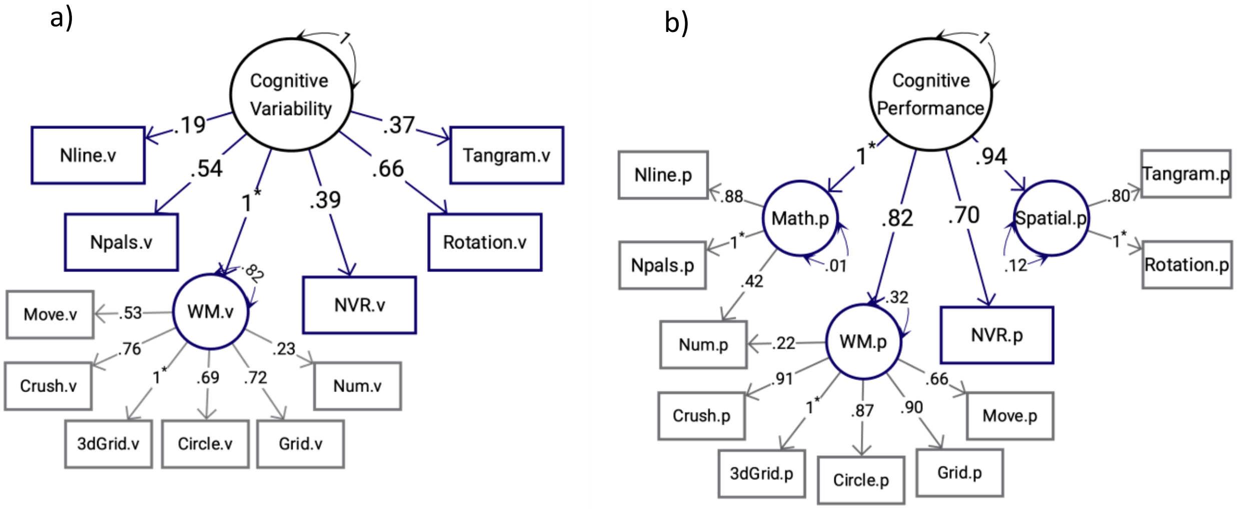

Supplementary Figure 5 The factor structure and standardized loadings for the best fitting CFA for ***a)*** *cognitive variability* and ***b)*** *cognitive mean performance*. Variability measures (denoted with '.v') are interindividual DSEM variance factor scores while performance measures (denoted with '.p') is the mean correct level per task. An asterisk indicates the path was fixed to one, therefore the unstandardized value (i.e., 1) is shown. See SI table 3 for model fit indices.

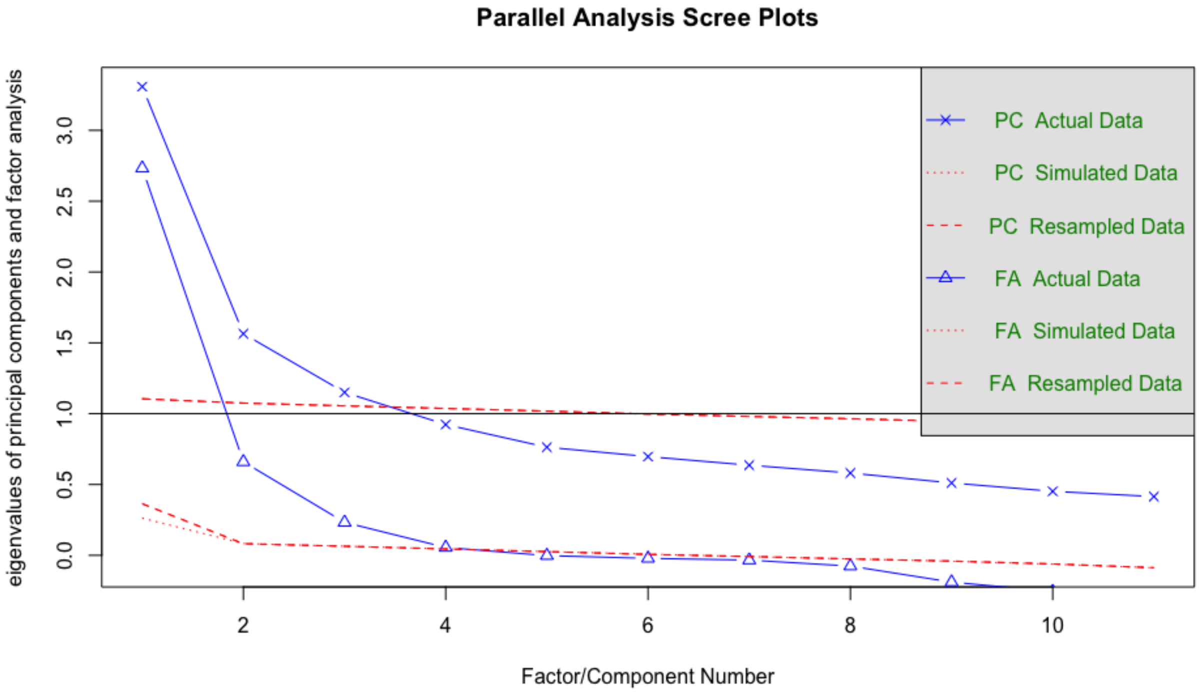

Supplementary Figure 6 A scree plot for cognitive *variability* measures. Triangles denote a factor from factor analysis, while the dashed red line is the result from parallel analysis.

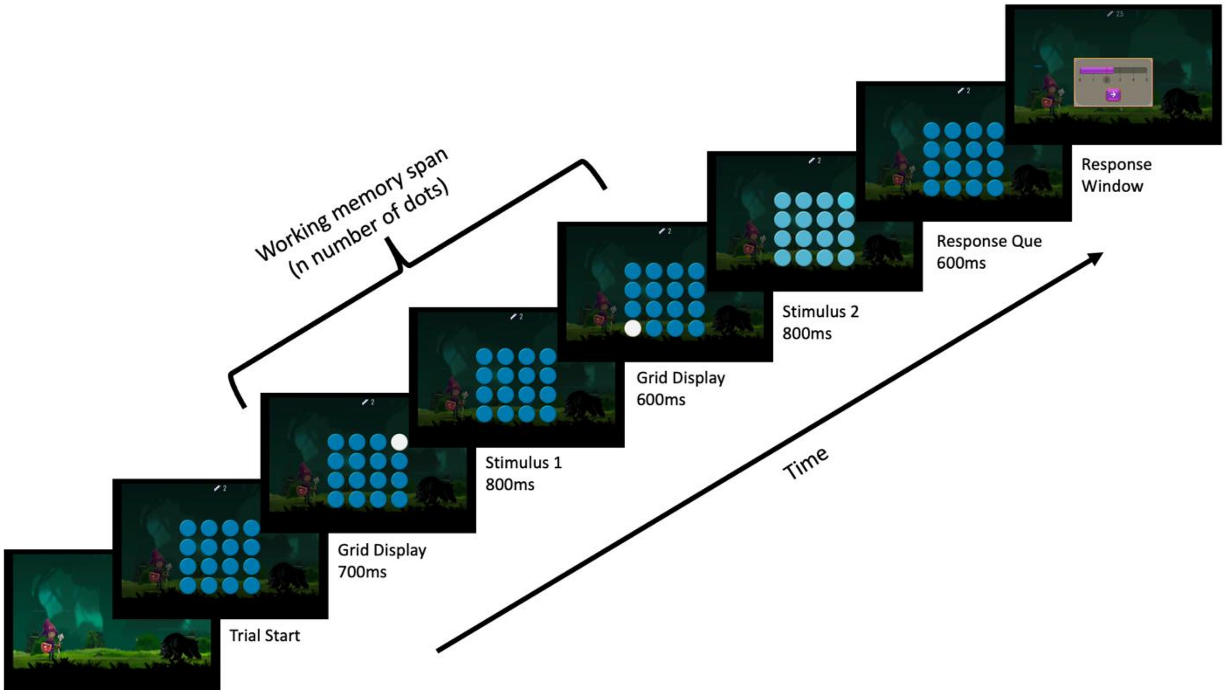

__Supplementary Figure 7__ An example of a visuospatial Working Memory Grid task span 2. Span refers to the number of dots that must be remembered, the span increases to increase trial difficulty.

Supplementary Table 1: DIC variance parameter model comparison.

Task	DIC Full Model	DIC variance constrained to zero	dDIC
WM_3dgrid (3DGrid)	-461276.26	-457423.98	3852.28
WM_circle (circle)	-381008.40	-378960.72	2047.6
WM_crush (crush)	-513901.28	-508919.89	4981.39
WM_grid (grid)	-972553.24	-961078.22	11475.02
WM_moving (move)	-198507.69	-198440.04	67.65
WM_numbers (num)	-99955.08	-98798.76	1156.32
Npals	3972961.99	3992464.11	19502.12
Numberline (nline)	3491346.66	3506689.49	15342.83
Non-verbal reasoning (nvr)	990719.55	993071.60	2352.05
Rotation (rot)	1623961	1633623.58	9662.58
Tangram (tan)	307540.24	308967.13	1426.89

SI Table 1 Caption: Deviance information criterion (DIC) model fit comparison table. Compares models where the residual variability parameter is either constrained to be equal across individuals (as a fixed effect) or allowed to differ (as a random effect). dDIC shows the subtracted model improvement from the unconstrained model to the variance constrained one. Across the board we see the random effect term for the residual variability parameter substantially improving model fit, see OSF (https://osf.io/z53an/) for model specification in MPLUS.

Supplementary Table 2: Trial variance reliability estimations.

Task	# subs	# Trials	Avg # trials	tv_rel.5	tv_rel.7	tv_rel.9
WM_3dgrid (3DGrid)	2608	203738	78.12	0.981	0.988	0.992
WM_circle (circle)	2608	155878	59.77	0.978	0.986	0.99
WM_crush (crush)	2608	243511	93.37	0.985	0.991	0.995
WM_grid (grid)	2608	430708	165.15	0.99	0.994	0.996
WM_moving (move)	2569	85640	33.34	0.971	0.98	0.986
WM_numbers (num)	2591	98300	37.94	0.952	0.972	0.984
Npals	2608	2398463	919.66	0.961	0.981	0.992
Numberline (nline)	2608	1833909	703.19	0.933	0.966	0.981
Non-verbal reasoning (nvr)	2608	548101	210.16	0.761	0.865	0.917
Rotation (rot)	2608	1032751	395.99	0.929	0.964	0.983
Tangram (tan)	2608	173128	66.38	0.593	0.76	0.858

SI Table 2 Caption: Trial variance reliability estimates are shown for three different scale reliability values (0.5 = tv_rel.5; 0.7 = tv_rel.7; 0.9 = tv_rel.9) derived from Du & Wang 2018. To be conservative we used the average number of trials for the number of assessments.

Supplementary Table 3: Confirmatory factor analysis fit indices

Task	Heywood	x^2	df	p	CFI	SRMR	RMSEA	Satorra-Bentler scaling factor
Model1_Var	False	1058.4	44	< .0001	0. 83	0.070	0.094 [0.089, 0.099]	1.12
Model1_mPerf	False	2339.3	44	<.0001	0. 89	0.057	0.141 [0.137, 0.146]	1.56
Model2_Var	True	506.6	40	<.0001	0.92	0.057	0.067 [0.062, 0.072]	1.12
Model2_mPerf	False	562.2	40	<.0001	0.98	0.027	0.071 [0.066, 0.076]	1.56
Model3_Var	False	517.0	43	<.0001	0.92	0.052	0.065 [0.060, 0.070]	1.12
Model3_mPerf	NA	NA	NA	NA	NA	NA	NA	NA

SI Table 3 Caption: Model 1 is a single factor solution, Model 2 is an a priori hierarchical subdomain (Math, WM, Spatial) model, while Model 3 is a hierarchical WM-only subdomain model (see SI Fig 2 for the specified CFA models). Var is short for cognitive variability measures while 'Perf' is short for mean performance measures. Heywood indicates there was a heywood case with negative variance, while NA indicates we did not attempt to fit the model as a prior model was adequate. SI Fig 5a shows the loadings of the best fit of cognitive variability (Model 3), while SI Fig 5b shows the best fit for mean performance (Model 2). Inference was based on RMSEA cutoff's of < .08 and CFI cutoffs of > .95; see Methods for further details.

Supplementary Table 4: Exploratory factor analysis loadings.

Task	Factor 1 (Working Memory)	Factor 2 (Math Reasoning)	Factor 3 (Tangram)
WM_3dgrid (3DGrid)	0.76	-0.01	-0.01
WM_circle (circle)	0.71	-0.03	-0.02
WM_crush (crush)	0.74	0.02	0.02
WM_grid (grid)	0.69	0.03	0.05
WM_moving (move)	0.57	-0.05	-0.05
WM_numbers (num)	0.20	-0.02	0.11
Npals	0.03	0.49	0.31
Numberline (nline)	0.08	0.36	-0.26
Non-verbal reasoning (nvr)	-0.02	0.65	-0.12
Rotation (rot)	0.17	0.42	0.21
Tangram (tan)	0.07	-0.02	0.63
